# Multimodal hybrid convolutional neural network based brain tumor grade classification

**DOI:** 10.1186/s12859-023-05518-3

**Published:** 2023-10-10

**Authors:** A. Rohini, Carol Praveen, Sandeep Kumar Mathivanan, V. Muthukumaran, Saurav Mallik, Mohammed S. Alqahtani, Amal Al-Rasheed, Ben Othman Soufiene

**Affiliations:** 1 Department of Computer Science and Engineering, Anil Neerukonda Institute of Technology and Sciences, Vishakapatnam, Andhra Pradesh 531162 India; 2 Department of Electronics and Communication Engineering, SSM Institute of Engineering and Technology, Dindigul, Tamilnadu India; 3 School of Computing Science and Engineering, Galgotias University, Greater Noida, 203201 India; 4 Department of Mathematics, College of Engineering and Technology, SRM Institute of Science and Technology, Kattankulathur, Tamilnadu 603203 India; 5 Department of Environmental Health, Harvard T H Chan School of Public Health, Boston, MA 02115 USA; 6 Department of Pharmacology and Toxicology, The University of Arizona, Tucson, AZ 85721 USA; 7 Radiological Sciences Department, College of Applied Medical Sciences, King Khalid University, 61421 Abha, Saudi Arabia; 8 BioImaging Unit, Space Research Centre, University of Leicester, Michael Atiyah Building, Leicester, LE1 7RH UK; 9 Department of Information Systems, College of Computer and Information Sciences, Princess Nourah bint Abdulrahman University, P.O. Box 84428, 11671 Riyadh, Saudi Arabia; 10 PRINCE Laboratory Research, ISITcom, Hammam Sousse, University of Sousse, 4000 Sousse, Tunisia

**Keywords:** Tumor classification, Magnetic resonance image, Deep learning, Transfer learning, Customized CNN, VGG19

## Abstract

An abnormal growth or fatty mass of cells in the brain is called a tumor. They can be either healthy (normal) or become cancerous, depending on the structure of their cells. This can result in increased pressure within the cranium, potentially causing damage to the brain or even death. As a result, diagnostic procedures such as computed tomography, magnetic resonance imaging, and positron emission tomography, as well as blood and urine tests, are used to identify brain tumors. However, these methods can be labor-intensive and sometimes yield inaccurate results. Instead of these time-consuming methods, deep learning models are employed because they are less time-consuming, require less expensive equipment, produce more accurate results, and are easy to set up. In this study, we propose a method based on transfer learning, utilizing the pre-trained VGG-19 model. This approach has been enhanced by applying a customized convolutional neural network framework and combining it with pre-processing methods, including normalization and data augmentation. For training and testing, our proposed model used 80% and 20% of the images from the dataset, respectively. Our proposed method achieved remarkable success, with an accuracy rate of 99.43%, a sensitivity of 98.73%, and a specificity of 97.21%. The dataset, sourced from Kaggle for training purposes, consists of 407 images, including 257 depicting brain tumors and 150 without tumors. These models could be utilized to develop clinically useful solutions for identifying brain tumors in CT images based on these outcomes.

## Introduction

In the realm of medical image processing, the ability to classify brain tumor images holds immense importance. This capability assists medical professionals in making precise diagnoses and formulating effective treatment plans. Magnetic resonance imaging (MRI) stands out as one of the primary imaging technologies used to examine brain tissue [1]. Nevertheless, the current gold standard for diagnosing and classifying brain tumors in medical practice remains histopathological examination of biopsy specimens. However, this approach is fraught with challenges—it is laborious, time-consuming, and susceptible to human errors. These limitations underscore the urgency of developing a fully automated technique for the multi-classification of brain tumors based on deep learning [2]. Over recent years, the medical image classification field has garnered significant attention, with convolutional neural networks emerging as the most widely employed neural network model for tackling image classification tasks [3]. A brain tumor represents an aberrant tissue where cells proliferate rapidly and uncontrollably, leading to tumor growth. Deep learning has been proposed as a solution to the challenges associated with brain tumor recognition and treatment. Notably, segmentation approaches have been instrumental in isolating abnormal tumor regions within the brain. For brain tumor-related tasks, reliable advanced artificial intelligence and neural network classification methods can play a pivotal role in early disease detection [4]. In recent times, the field of medical science has witnessed remarkable growth and success, largely driven by technological advancements. The transformative power of technology is revolutionizing the medical field. Artificial intelligence, a discipline focused on creating machines capable of independent learning without human intervention, has played a crucial role in this transformation. Machine learning has enabled the construction of computers that can emulate human thought processes and learn from experience. Real-world applications now leverage natural language understanding and deep learning techniques to address a wide array of challenges, including optimizing complex systems, categorizing vast digital datasets, identifying patterns, and advancing the development of self-driving cars [5]. Recent advancements in medical imaging, made possible by deep learning, have significantly improved the ability to diagnose a wide range of diseases. The most common and widely used machine learning method for visual learning and image recognition is the CNN architecture [6]. The human brain is safeguarded by a sturdy skull. Any expansion within this confined space can lead to significant complications. When tumors, whether normal or malignant, develop within the skull, they cause an increase in intracranial pressure. Consequently, permanent brain damage and even mortality can occur. Globally, approximately 700,000 people are affected by brain tumors, with an estimated 86,000 new cases diagnosed in 2019. In response to this challenge, researchers and scientists have been diligently working to develop more advanced tools and methods for the early detection of brain tumors [7].

The human brain, one of the most complex organs in the body, is composed of billions of individual cells that interact with each other. It is believed that the progression of unregulated cell division is responsible for the development of brain tumors. This process results in the formation of abnormal cell growth within or around the brain, which can be further categorized as either normal or malignant. The likelihood of developing a brain tumor during one's lifetime continues to increase. Abnormal cell growth, which affects both healthy and unhealthy cells, can impair the brain's proper functioning. According to a research organization focused on cancer in the UK, 5250 people die each year from brain-related conditions. Furthermore, the World Health Organization (WHO) reports that brain tumors account for less than 2% of all human cancers. The current WHO classification for brain tumors is exclusively based on histopathology, which significantly limits its applicability in clinical settings [8].

Our current way of life wouldn't be possible without the contributions of information technology. The field that focuses on creating machines capable of autonomous learning, without human intervention, is known as artificial intelligence. Thanks to machine learning, people can now develop computers that can think and learn from experiences much like humans do. Natural language processing and deep learning play integral roles in numerous real-world applications today. For instance, they are employed to solve complex optimization problems, classify vast volumes of digital data by identifying relevant patterns, and enable self-driving cars. Deep learning, a subfield of machine learning, involves inputting information into a deep learning model, which then autonomously learns without human interference [9]. The process of diagnosing a brain tumor and determining its grade is often labor-intensive and time-consuming. Typically, patients request an MRI when the brain tumor reaches a certain size and causes various troublesome symptoms. After reviewing the patient's brain scans, if there is suspicion of a tumor, the next step is to perform a brain biopsy. Unlike magnetic resonance imaging, the biopsy procedure is invasive, and in some cases, the results may not be clear for up to a month. Professionals working with MRI employ techniques such as perfusion and biopsy to grade tumors and confirm their findings. It's worth noting that, in addition to biopsies, several innovative procedures for grading brain tumors have been developed in recent years [10]. This paper presents a novel CNN-based model for classifying brain tumors into two categories: malignant and normal. The CNN model is trained and developed using a large dataset. To enhance the accuracy of the proposed model, preprocessing techniques such as normalization and data augmentation are implemented on the dataset. Therefore, automated systems like this one are valuable for saving time and improving efficiency in clinical institutions. The proposed brain tumor grade classification model consists of five sections: “Introduction” section deals with the various types of tumors, different brain tumor grades, and their diagnosing tools. “Related work” section discusses state-of-the-art methods for brain tumor grade classification and their classification techniques. “Materials and methods” section illustrates the utilization of the dataset and the proposed model's classification architectures. “Experimental results and discussion” section discusses the outcomes of the proposed brain tumor classification hyperparameters and compares them to the outcomes of state-of-the-art methods. “Conclusion and future work” section summarizes the proposed work, concludes, and outlines the scope of future work in brain tumor grade classification.

## Related work

Our current way of life wouldn't be possible without the contributions of information technology. The field that focuses on creating machines capable of autonomous learning, without human intervention, is known as artificial intelligence. Thanks to machine learning, people can now develop computers that can think and learn from experiences much like humans do. Natural language processing and deep learning play integral roles in numerous real-world applications today. For instance, they are employed to solve complex optimization problems, classify vast volumes of digital data by identifying relevant patterns, and enable self-driving cars. Deep learning, a subfield of machine learning, involves inputting information into a deep learning model, which then autonomously learns without human interference [11]. The process of diagnosing a brain tumor and determining its grade is often labor-intensive and time-consuming. Typically, patients request an MRI when the brain tumor reaches a certain size and causes various troublesome symptoms. After reviewing the patient's brain scans, if there is suspicion of a tumor, the next step is to perform a brain biopsy. Unlike magnetic resonance imaging, the biopsy procedure is invasive, and in some cases, the results may not be clear for up to a month. Professionals working with MRI employ techniques such as perfusion and biopsy to grade tumors and confirm their findings. It's worth noting that, in addition to biopsies, several innovative procedures for grading brain tumors have been developed in recent years [12].

We present a novel two-stage graph coarsening method rooted in graph signal processing and its application within the GCNN architecture. In the first coarsening stage, we employ graph wavelet transform (GWT)-based features to construct a coarsened graph while preserving the original graph's topological properties. This is achieved through the use of the graph wavelet transform. In the second phase, the coarsening problem is treated as an optimization challenge. At each level, we obtain the reduced Laplacian operator by restricting the initial Laplacian operator to a predefined subspace that maximizes topological similarity. This restriction of the initial Laplacian operator to the specified subspace yields the reduced Laplacian operator for each level. The results demonstrate that, whether used for general coarsening or as a pooling operator within the GCNN architecture, the proposed coarsening method outperforms current best practices [13].

The algorithms encompass graph embedding and graph regularization models, with their primary aim being to leverage the local geometry of data distribution. Graph Convolutional Networks (GCN), which successfully extend Convolutional Neural Networks (CNNs) to handle graphs with arbitrarily defined structures by incorporating Laplacian-based structural information, represent one of the most prominent approaches in Multiple-Source Self-Learning (MSSL) [14]. For the classification of various types of brain tumors, including both normal and abnormal magnetic resonance (MR) images, we propose the use of a differentially deep Convolutional Neural Network (CNN) model. This model generates additional differential image features from the original CNN feature maps by employing divergence operators within the differential deep-CNN architecture. The differential deep-CNN model offers several advantages, including the ability to analyze pixel direction through contrast calculations and the capability to categorize a large image database with high accuracy and minimal technical issues. As a result, the suggested strategy delivers outstanding overall performance [15]. Utilizing computer-aided mechanisms instead of traditional manual diagnostic procedures allows for superior results. Typically, this involves feature extraction using a Convolutional Neural Network (CNN), followed by data classification using a fully connected network. The proposed work employs "deep neural networks" and incorporates a CNN-based model to classify MRI results as either "tumor detected" or "tumor not detected." The model achieves an average accuracy of 96.08%, with an f-score value of 97.3 on average [16].

Due to the risk of overfitting in the development of deep Convolutional Neural Networks, it is rare for small datasets to benefit from such models. We propose a modified deep neural network and apply it to a small dataset. Additionally, we discuss the implications of our findings. Our approach entails using the VGG16 architecture with CNN as the classifier. We evaluate our proposed method's performance by testing it on the VGG16 dataset and measuring its precision, recall, and F-score to assess its effectiveness [17].

A novel hybrid model, combining the VGG16 Convolutional Neural Network (CNN) and Neural Autoregressive Distribution Estimation (NADE), referred to as VGG16-NADE, was developed. The study encompassed a dataset comprising 3,064 MRI images of brain tumors, categorized into three groups. To classify the T1-weighted contrast-enhanced MRI images, we employed the VGG16-NADE hybrid framework and compared it to other methods. The results indicated that the developed hybrid VGG16-NADE model outperforms other models in terms of accuracy, specificity, sensitivity, and the F1 score. The suggested hybrid VGG16-NADE model achieved a prediction accuracy of 96.01%, precision of 95.72%, recall of 95.64%, F-measure of 95.68%, a receiver operating characteristic (ROC) of 0.91, an error rate of 0.075, and a Matthews correlation coefficient (MCC) of 0.3564 [18].

The findings of this study demonstrate that an MRI can effectively detect brain tumors in two steps. The initial step involves an image enhancement procedure utilizing clip limit adaptable histogram equalization (CLAHE) to segment the brain MRI. The subsequent step entails identifying the type of brain tumor depicted in the MRI, employing the Visual Geometry Group-16 Layer (VGG-16) model. In specific instances, CLAHE was employed, such as applying it to the FLAIR image to enhance the green color and using it in the Red, Green, and Blue (RGB) color space. The experimental results revealed the highest performance, achieving an accuracy of 90.37%, precision of 90.22%, and recall of 87.61%. Notably, both the CLAHE approach in the RGB channel and the VGG-16 model consistently distinguished oligodendroglioma subclasses in RGB enhancement, with a precision rate of 91.08% and a recall rate of 95.97% [19].

The process of image segmentation and the transformation into models depend on their functionality, which, in turn, relies on various algorithms and the degree of technological advancement in application techniques. Through segmentation techniques, it is now possible to create three-dimensional models of a patient's body, enabling study without risking the patient's life. In this study, a combination of two methods for addressing segmentation challenges is discussed, followed by an explanation of how these methods are integrated into a hybrid algorithmic structure. Convolutional neural networks (CNNs), also known as active contour and deep multi-planar, are utilized to convert DICOM medical images (Digital Imaging and Communication Systems in Medicine) into 3D models. The data from the active contour method serves as input for deep learning [20]. In the field of medical image processing, the author proposes a Convolutional Neural Network Database Learning with Neighboring Network Limitation (CDBLNL) approach for brain tumor image classification. The suggested system architecture employs multilayer-based metadata learning, incorporating a CNN layer to provide reliable data. The approach uses two datasets (BRATS and REMBRANDT) and achieves a 97.2% accuracy result in brain MRI categorization [21]. This study suggests using cervigram images for cervical cancer detection. The Associated Histogram Equalization (AHE) approach enhances cervical image edges, while the finite ridgelet transform creates a multi-resolution image. This modified multi-resolution cervical image provides ridgelets, gray-level run-length matrices, moment invariants, and an enhanced local ternary pattern. A feed-forward, backward-propagation neural network trains and tests these derived features to identify cervical images as normal or abnormal. Morphological methods are employed on aberrant cervical images to detect and segment carcinoma. The cervical cancer detection method demonstrates 98.11% sensitivity, 98.97% specificity, 99.19% accuracy, a PPV of 98.88%, an NPV of 91.91%, an LPR of 141.02%, an LNR of 0.0836, 98.13% precision, 97.15% FPs, and 90.89% FNs [22]. Compared to these state-of-the-art classifiers, the author's suggested Particle Swarm Optimization (PSO) technique, applied to selected characteristics of the NSL-KDD dataset, reduced the false alarm rate while increasing the detection rate and accuracy of the IDS. Measures of IDS performance such as accuracy, precision, false-positive rate, and detection rate are included in this analysis. Out of a total set of 41 characteristics, 10 were selected, which exhibited low computational complexity, 99.32% efficiency, and a 99.26% detection rate in the experiment [23]. In order to find the best feature subsets in the NSL-KDD dataset, the author suggests a new feature selection approach based on a genetic algorithm (GA). To further improve DR (Detection Rate) and ACC (Accuracy), hybrid classification utilizing logistic regression (LR) and decision tree (DT) has been performed. This study optimized the selected optimal features by applying and comparing the results of multiple meta-heuristic techniques. According to the data, the Grey Wolf Optimization (GWO) method achieves the highest accuracy (99.44%) and detection rate (99.36%) when only 20% of the original characteristics are used [24]. "For detecting tumors in MRI scans, a modified version of the pre-trained InceptionResNetV2 model is utilized. Once a tumor is located, its stage (which may be glioma, meningioma, or pituitary cancer) is determined using a combination of InceptionResNetV2 and Random Forest Tree (RFT). To address the limited size of the dataset, we employ Cyclic Generative Adversarial Networks (C-GAN). The experimental findings indicate that the proposed models for tumor detection and classification are highly accurate (99% and 98%, respectively) [25]. Author used a smart combination of deep learning techniques to reduce unwanted speckles in breast ultrasound pictures. We first improved the image contrast, then made fine details clearer with special filters. To fix overly sharp areas, we applied a filter, and we also added a feature to protect important edges in the pictures. After training our model a hundred times, we achieved excellent results. Both the error rate and the number of false identifications were less than 1.1%. This shows that our model, called LPRNN, is good at reducing speckles without losing important parts of the images [26]. Author used two pre-trained CNN models, VGG16 and VGG19, to extract features from the data. Then, we applied a correntropy-based learning strategy with the extreme learning machine (ELM) to identify the most important features. We enhanced these features further using a robust covariant technique based on partial least squares (PLS) and combined them into one matrix. Finally, we used this combined matrix as input for the ELM to classify the data. To test our method, we ran experiments on the BraTS datasets. The results were impressive, with accuracy rates of 97.8%, 96.9%, and 92.5% for BraTs2015, BraTs2017, and BraTs2018, respectively [27].

## Materials and methods

While there has been a significant amount of research on brain tumors, only a limited amount of work has been published comparing four deep learning models—VGG16, VGG19, ResNet101, and DenseNet201—in order to draw conclusions regarding the distinctions between tumor types. Next, we generate accuracy, loss, and learning curve graphs, and establish testing procedures to visualize and compare the performance of these models.

### Materials

**Fig. 1:**
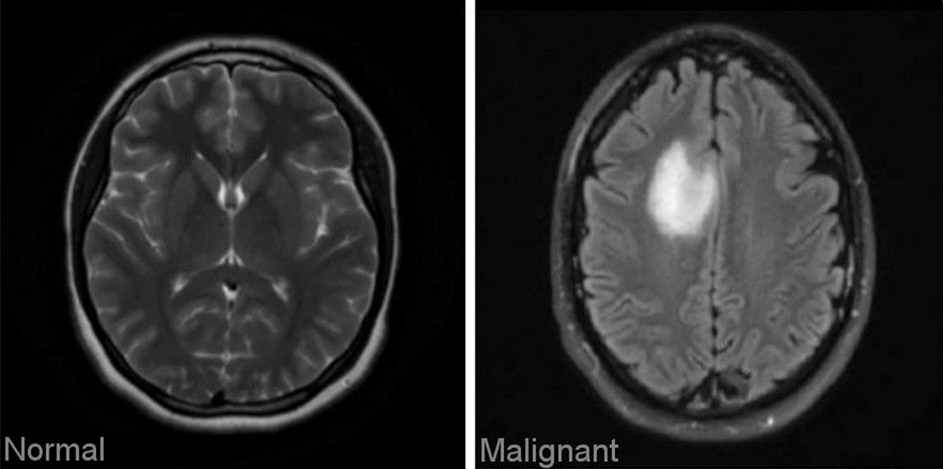
Brain tumor images from dataset (sample)

The proposed method utilizes the publicly available dataset "Brain MRI Images for Brain Tumor Identification," created by Abd El Kader on February 15, 2020, which can be accessed at (https://www.kaggle.com/navoneel/brain-mri-images-for-brain-tumor-detection/). The dataset comprises two main subsets: brain tumor images (n = 257) and normal images (n = 150), all with dimensions of 467 × 586 × 3. This dataset is divided into two sections: one designated as the training section and the other as the testing section. Table 1 provides an overview of the dataset categories, while Table 2 and Fig. 1 showcase sample images from the dataset.

**Table 1:** Outcome comparison of state-of-the-art methods

Author	Approach	Objective	Outcome
Wadhah Ayadi [11]	Deep CNN	We exploited CNN for the problem of brain tumor classification. The proposed model, which contains various layers, aims to classify MRI brain tumor	The proposed model achieved higher accuracy of 97.02% and outperformed the various previous works
Xinyu Lei [12]	Hybrid dilated CNN	Proposed a dilated CNN model, which is built by replacing the convolution kernels of traditional CNN by dilated convolution kernels, and then, the dilated CNN model is tested on the Mnist handwritten digital recognition data set	The dilated CNN model reduces the training time by 12:99% and improves the training accuracy by 2:86% averagely, compared with the dilated CNN model
Himanshu Padole [13]	Graph CNN, graph wavelet transforms	A novel two-stage graph coarsening method rooted in graph signal processing and its application in the GCNN architecture	The proposed model achieved higher accuracy of 99.30% and outperformed the various previous works
Sichao Fu [14]	Graph-based semi-supervised learning, manifold assumption based SSL algorithms	The spectral graph Hessian convolutions is a combination of the Hessian matrix and the spectral graph convolutions	HesGCN can learn more efficient data features by fusing the original feature information with its structure information based on Hessian
Isselmou Abd El Kader [15]	differential deep-CNN	differential deep convolutional neural network model to classify different types of brain tumor, including abnormal and normal MR images	The experimental results showed that the proposed model achieved an accuracy of 99.25%
Chirodip Lodh Choudhury [16]	Extracting features through a CNN	Deep neural network and incorporates a CNN based model to classify the MRI as “detected” or “not detected”	The model captures a mean accuracy score of 96.08% with f-score of 97.3
Anushka Singh [17]	Deep CNN	The proposed deep learning method which is used to classify Brain tumor types. Our method is based on VGG16 architecture with CNN as the classifier	An accuracy of above 93% along with high precision, recall and F-score was achieved
Saran Raj Sowrirajan [18]	VGG16 and Neural Autoregressive Distribution Estimation	Analyzing MRI through deep learning models is the most prevalent and accurate method of early cancer detection	Prediction accuracy of the proposed hybrid VGG16-NADE is 96.01%, precision 95.72%, recall 95.64%, F-measure 95.68%, ROC 0.91, error rate 0.075, and the MCC 0.3564
Suci Aulia [19]	Clip Limit Adaptive Histogram Equalization	Automatic brain tumor detection technology to identify the presence of a tumor in the brain without requiring human intervention	Highest performance with Acc, Pr, Recall, respectively 90.37%, 90.22%, 87.61%
Rafeek Mamdouh [20]	CNN, Active Contour & Deep Multi-Planar	Combination of two fields of solving segmentation problem to convert through the workflow of a hybrid algorithm structure CNN	System achieved a high result of segmentation, each image contains edges and image size = 256p × 256p, using Active Contour Model can generate multiple results for output, by resizing the threshold frames and gray-scale image and adjustment control, and generate a multi 3D model as output by changing the Histogram and Gaussian equation

**Table 2:** Description of the brain tumor dataset

No	Brain tumor	Training images (80%)	Testing images (20%)
1	Normal	120	30
2	Malignant	205	52

#### Brain tumor prediction using pretrained CNN models

**Fig. 2:**
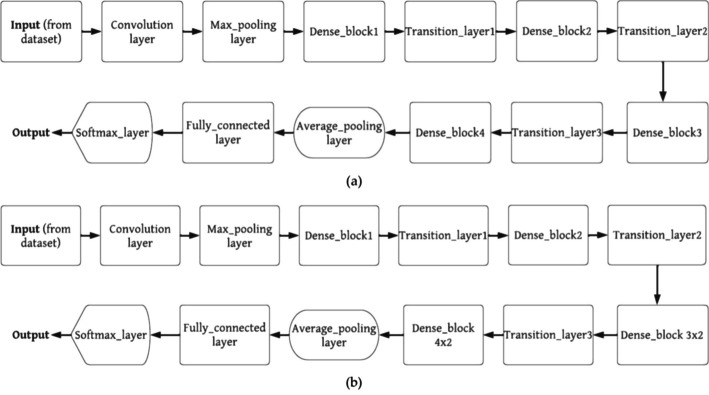
Function blocks of DenseNet101, and DenseNet201

**Fig. 3:**
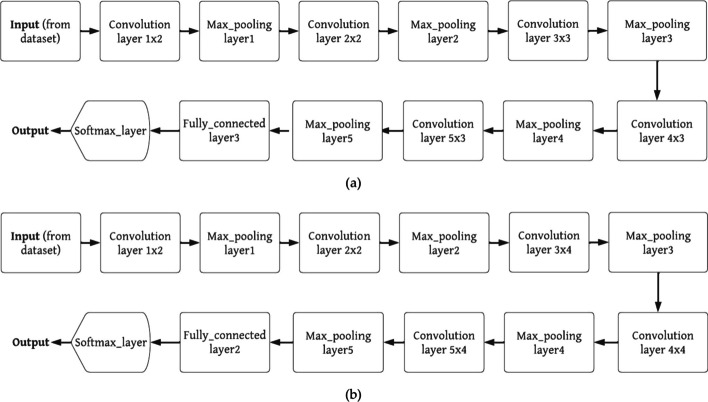
Function blocks of VGG16, and VGG19

Convolutional neural network (CNN) models have repeatedly demonstrated their ability to produce high-quality results in various healthcare research and application areas. However, building these pretrained CNN models from scratch for predicting neurological conditions using computed tomography (CT) data has always been a challenging task [28]. These pretrained models are inspired by the concept of "transfer learning," where a deep learning model trained on a large dataset is employed to address a problem in a smaller dataset [29]. Transfer learning leverages the idea that one dataset can be used to train another, eliminating the need for a large dataset and the lengthy training periods often required by many deep learning models. In this paper, four deep learning models are utilized: DenseNet101, DenseNet201, VGG16, and VGG19. These models were initially trained on ImageNet and subsequently fine-tuned using examples of cancerous tissue. After pretraining, a fully connected layer is added to the last layer [30]. Tables 3 and 4 provide architectural descriptions and functional block details for each design. Table 4 presents the parameters, while Figs. 2 and 3 illustrate the functional block diagrams of VGG16, DenseNet101, VGG19, and DenseNet201.

**Table 3:** The detailed architecture of DenseNet101 and DensNet201

No. of Layer	Size of the output	DenseNet101	DenseNet201
Convolution block	Size of 224 × 224	12 × 12 with stride of 2	12 × 12 with stride of 2
Pooling_layer	Size of 112 × 112	6 × 6 max_pool with stride of 2	6 × 6 max_pool with stride of 2
Dense_block 1	Size of 112 × 112	3 × ((conv_1 × 1), (conv_3 × 3))	3 × ((conv_1 × 1), (conv_3 × 3))
Transition_layer 1	Size of 112 × 112	conv_1 × 1	conv_1 × 1
	Size of 56 × 56	average_pool 2 × 2 with stride of 2	average_pool 2 × 2 with stride of 2
Dense_block 2	Size of 56 × 56	6 × ((conv_1 × 1), (conv_3 × 3))	6 × ((conv_1 × 1), (conv_3 × 3))
	Size of 56 × 56	conv_1 × 1	conv_1 × 1
Transition_layer 2	Size of 56 × 56	7 × 7 with stride of 2	7 × 7 with stride of 2
	Size of 28 × 28	3 × 3 max_pool with stride of 2	3 × 3 max_pool with stride of 2
Dense_block 3	Size of 28 × 28	6 × ((conv_1 × 1), (conv_3 × 3))	6 × ((conv_1 × 1), (conv_3 × 3))
	Size of 28 × 28	conv_1 × 1	conv_1 × 1
Transition_layer 3	Size of 28 × 28	average_pool 2 × 2 with stride of 2	average_pool 2 × 2 with stride of 2
	Size of 14 × 14	12 × ((conv_1 × 1), (conv_3 × 3)	12 × ((conv_1 × 1), (conv_3 × 3)
Dense_block 4	Size of 14 × 14	conv_1 × 1	conv_1 × 1
	Size of 14 × 14	average_pool 2 × 2 with stride of 2	average_pool 2 × 2 with stride of 2
Transition_layer 4	Size of 14 × 14	24 × ((conv_1 × 1), (conv_3 × 3)	48 × ((conv_1 × 1), (conv_3 × 3)
	Size of 7 × 7	conv_1 × 1	conv_1 × 1
Dense_block 5	Size of 7 × 7	average_pool 2 × 2 with stride of 2	average_pool 2 × 2 with stride of 2
	Size of 7 × 7	16 × ((conv_1 × 1), (conv_3 × 3)	32 × ((conv_1 × 1), (conv_3 × 3)
Classification_layer	Size of 1 × 1	global_average_pool 7 × 7	global_average_pool 7 × 7
	1000	Softmax layer_fully connected	Softmax layer_fully connected

**Table 4:** The detailed architecture of Visual Geometry Group-16 and Visual Geometry Group-19

No. of layer	Size of the output	Visual geometry group-16	Visual geometry group-19
Convolution block 1	Size of 224 × 224	conv 2D × 2	conv 2D × 2
	Size of 112 × 112	2D max_pooling layer	2D max_pooling layer
Convolution block 2	Size of 112 × 112	conv 2D × 2	conv 2D × 2
	Size of 56 × 56	2D max_pooling layer	2D max_pooling layer
Convolution block 3	Size of 56 × 56	conv 2D × 3	conv 2D × 4
	Size of 28 × 28	2D max_pooling layer	2D max_pooling layer
Convolution block 4	Size of 28 × 28	conv 2D × 3	conv 2D × 4
	Size of 14 × 14	2D max_pooling layer	2D max_pooling layer
Convolution block 5	Size of 14 × 14	conv 2D × 3	conv 2D × 4
	Size of 7 × 7	2D max_pooling layer	2D max_pooling layer
Classification layer	4096	(softmax layer|fully connected layer) × 3	(softmax layer|fully connected layer) × 3

DenseNet101 comprises 10.2 million trainable parameters and includes one convolutional layer, one max pooling layer, three transition layers, one average pooling layer, one fully connected layer (FCL), and one Softmax layer. It also features four dense block layers, with the third and fourth dense blocks each containing one convolution layer with a stride of 1 × 1 and the third and fourth dense blocks each having a stride of 3 × 3, respectively [31]. The DenseNet201 model also consists of 10.2 million trainable parameters and consists of one convolution layer, one max pooling layer, three transition layers, one average pooling layer, one fully connected layer, and one softmax layer. Additionally, it incorporates four dense block layers, with the third and fourth dense block layers each housing two convolution layers with stride ratios of 1 × 1 and 3 × 3, respectively [32]. VGG16 boasts 138 million trainable parameters and includes thirteen convolutional layers, five max pooling layers, three fully connected layers, and one softmax layer.

### Methods

**Fig. 4:**
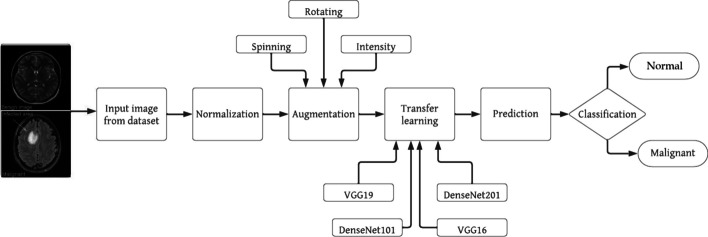
Proposed model operational block diagram

Figure 4 illustrates the proposed model for brain tumor identification. This model categorizes brain tumor images into two distinct categories: normal and malignant. To ensure numerical stability and enhance the performance of deep learning models, the dataset underwent a normalization pre-processing approach. The computed tomography images initially exist in either monochrome or grayscale formats, with pixel values ranging from 0 to 255. Normalizing these input images is a crucial step, as it significantly accelerates the training of deep learning models.

**Fig. 5:**
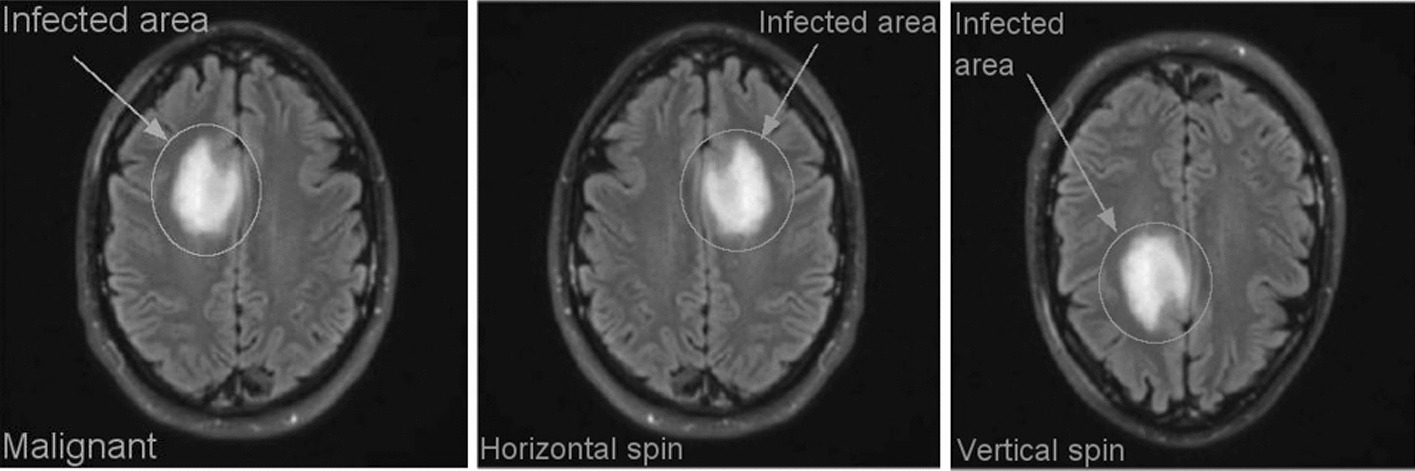
Different orientation of brain (malignant) tumor image

Having a substantial amount of data is essential when attempting to improve the performance of a deep learning model. However, access to these datasets often faces various constraints. Consequently, to overcome these challenges, data augmentation methods are employed to increase the total number of sample photos within the dataset. Various techniques for enhancing the data, including flipping, rotating, adjusting intensities, and zooming, are implemented. In Fig. 5, you can observe examples of the horizontal flipping technique as well as the vertical flipping approach.

**Fig. 6:**
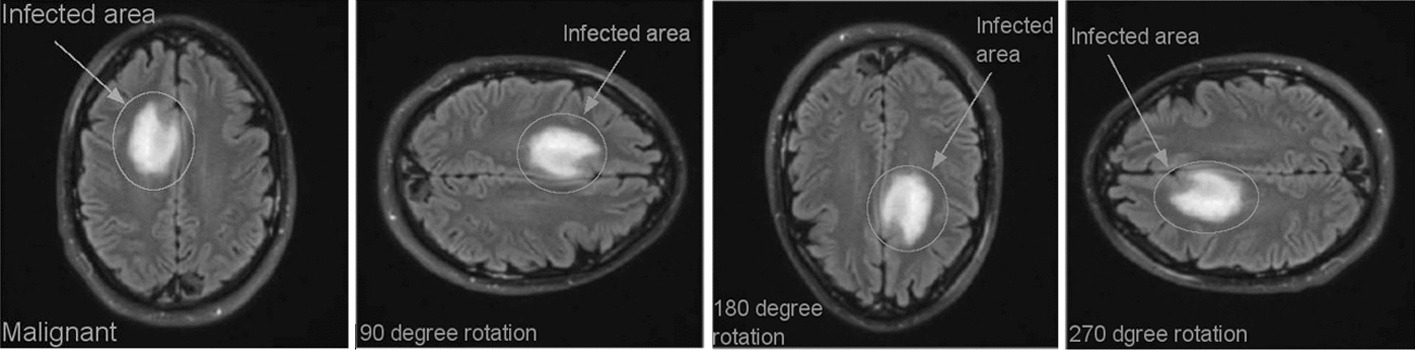
Different orientation (clock wise) of tumor image (data augmentation)

**Fig. 7:**
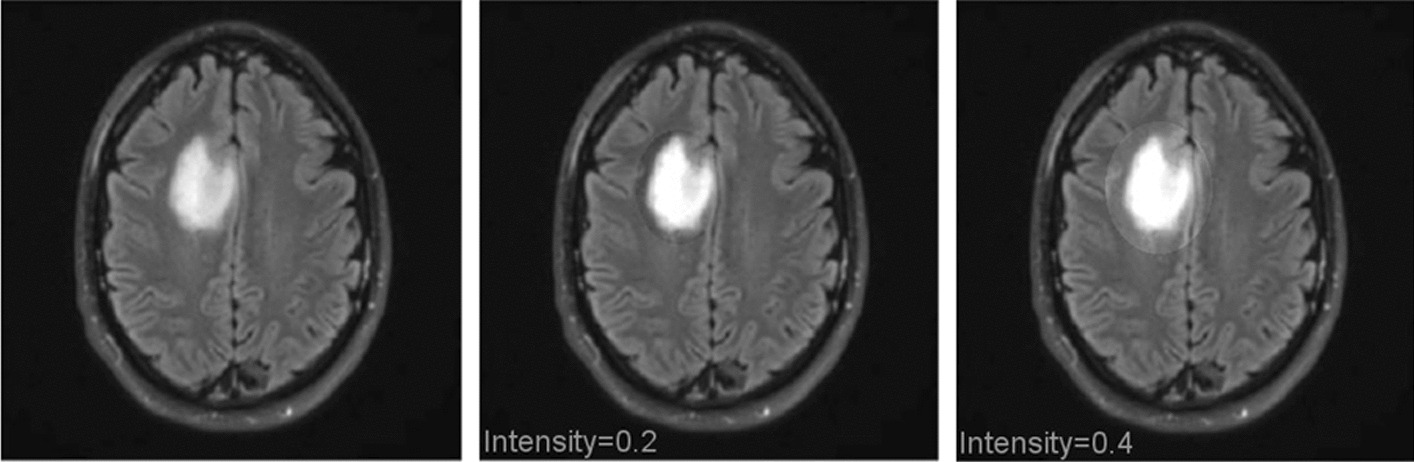
Intensity data augmentation of original image, intensity factor 0.2, and 0.4

Figure 6 depicts a rotation augmentation approach that is implemented in a clockwise direction by an angle of 90° each. Using intensity factor values such as 0.2 and 0.4 as examples, the intensity data augmentation technique that is illustrated in Fig. 7 is also applied to the image dataset.

Table 5 presents training images captured both before and after the augmentation process. Additionally, the input dataset exhibits an uneven distribution of classes. Data augmentation strategies are employed to address this identified imbalance issue. After applying these data augmentation techniques, the sample dataset for each class is increased by 70%, resulting in the dataset being expanded to a total of 2850 images.

**Table 5:** Sample images prior and post data augmentation

Tumor type	Total number of image—prior augmentation	Total number of images—post augmentation (70%)
Malignant	257	1800
Normal	150	1050

## Experimental results and discussion

An experimental study is being conducted to detect brain tumors from CT scans using four pretrained hybrid CNN models: VGG16, DenseNet 101, DenseNet 201, and VGG19. These hybrid classifiers were implemented using CT images from the brain tumor dataset. For training and testing, a total of 205 images were used for training, while 52 images were reserved for testing. The initial dimensions of the brain scans were reduced from 467 × 586 to 224 × 224 to facilitate transfer learning. The models were trained with a batch size of 16, determined through empirical methods. Each model underwent a total of 20 training epochs, with the learning rate determined empirically. The execution time of our study was longer due to the complexity and high frequency of layers in the network, which justified the good accuracy we obtained. The longer execution time in the current study can be attributed to the number of hidden layers, pooling layers, and batch sizes. It should be noted that training deeper networks requires more time than training shallower or simpler networks. Training was carried out using the Adam optimizer. Various performance indicators, including accuracy, precision, sensitivity, specificity, and the F2 score, were used to assess the performance of each model.

### Performance metrics

The following measurements have been used to evaluate the model that was suggested: Sensitivity, which refers to the percentage of true positives that can be identified without error; Specificity, which reflects the percentage of false negatives that are accurately identified; Precision can be defined as the ratio of correct positive forecasts to the total number of positive predictions, whereas accuracy refers to the proportion of true positives as well as true negatives. The Eqs. 1, 2, 3, 4, and 5 each have a parameter that is described by that equation.

1 $$Sensitivity = \frac{\alpha }{\alpha + \beta }$$

2 $$Specificity = \frac{\emptyset }{\emptyset + \mu }$$

3 $$Precision = \frac{\alpha }{\alpha + \mu }$$

4 $$Accuracy = \frac{\alpha + \emptyset }{{\alpha + \mu + \emptyset + \beta }}$$

5 $$F2 score = 2\frac{precsion \times recall}{{precsion + recall}}$$

6 $$Recall = \frac{\alpha }{\alpha + \beta }$$

where true positives (*α*) are the correctly classified positive cases, true negatives (*ø*) are the correctly classified negatives, false positives (*µ*) are the incorrectly classified positives, false negatives (*β*) are the incorrectly classified negatives.

### A comparison of the training results obtained from the several models

**Fig. 8:**
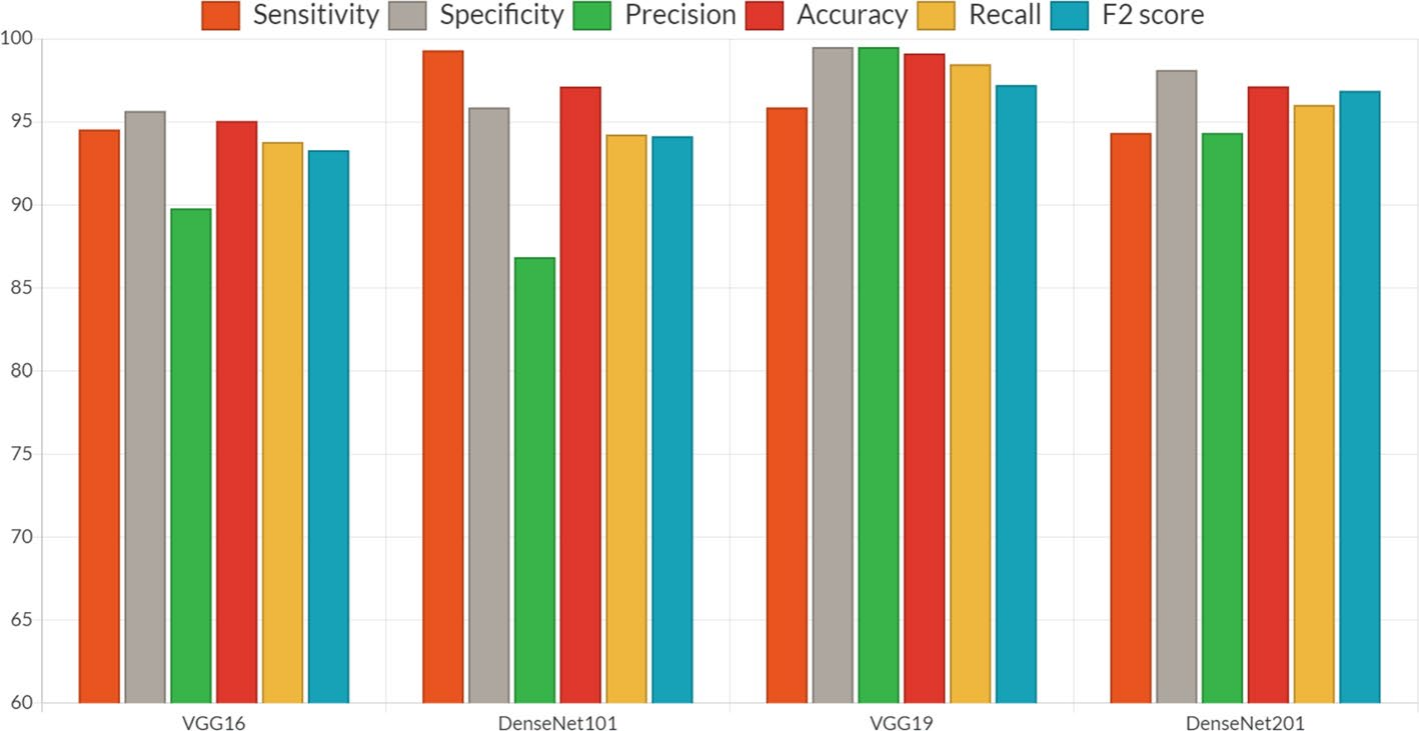
Parameters comparison of VGG19 and various CNN models

**Fig. 9:**
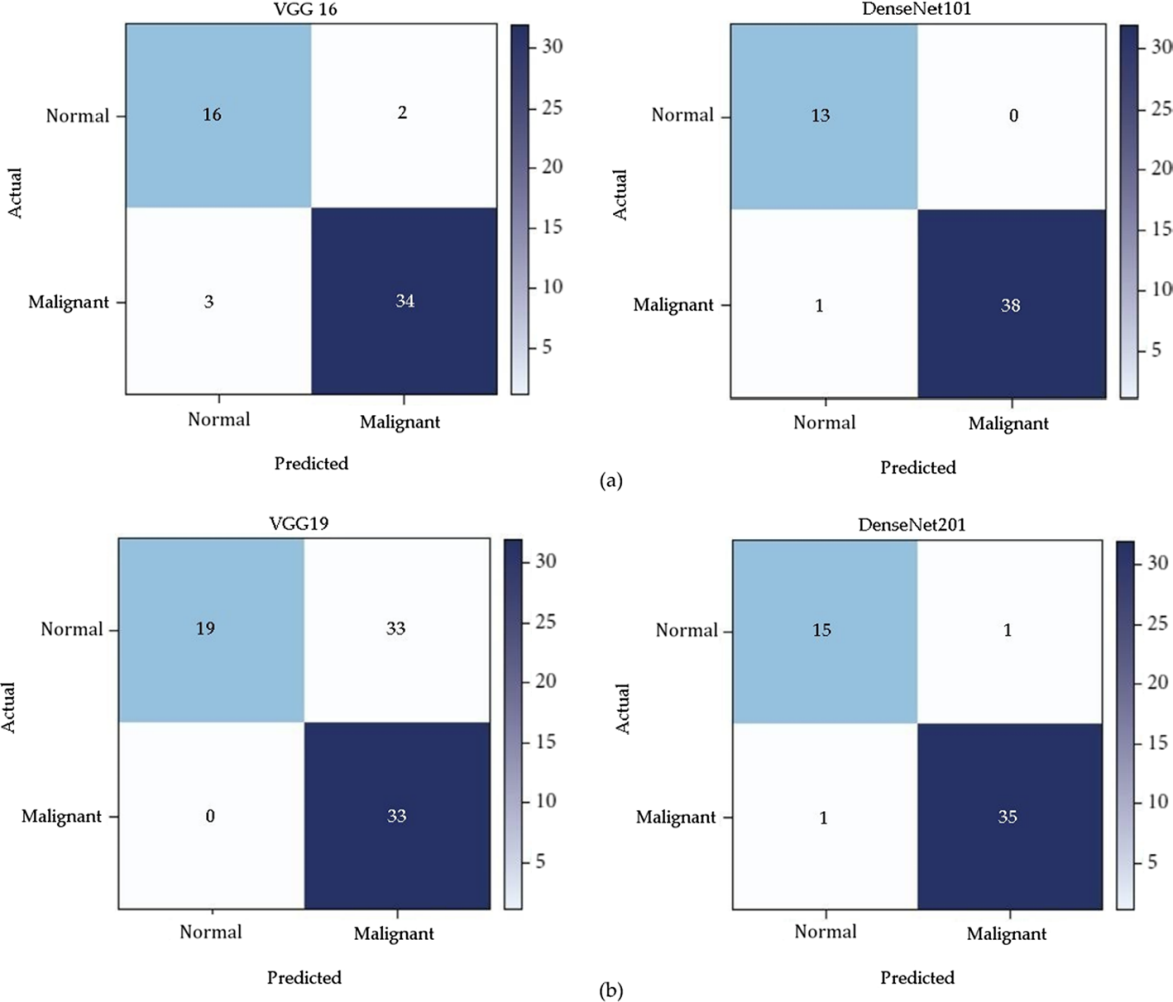
Confusion matrix for models: **a** VGG-16 and DenseNet101, **b** VGG-19 and DenseNet201 with batch size of 16

To obtain a range of performance parameters, four distinct models, each with a unique combination of epochs and batch sizes, are employed. These parameters encompass training loss, error rate, testing loss, and testing accuracy. Specifically, the four different models—VGG16, DenseNet 101, DenseNet 201, and VGG19—were each tested with 20 epochs and a batch size of 16. The training of these deep learning models is carried out using the Adam optimizer. Table 6 illustrates that among these models, VGG19 exhibits the highest performance during testing with a batch size of 16. It achieved a precision of 99.5%, sensitivity of 95.86%, specificity of 99.5%, an accuracy rate of 99.11%, and an F2 score of 97.21%. Furthermore, as shown in Table 7, VGG-19 outperforms the other models during the training phase, evidenced by its lower testing loss and the highest testing accuracy. VGG19 consists of 19 layers, which is similar in number to DenseNet101 and DenseNet201. It features approximately 8 million parameters, which is fewer than DenseNet101 and DenseNet201. While DenseNet101 and DenseNet201 share similar functionalities, DenseNet201 has a greater number of layers, resulting in longer processing times. After 20 iterations, the performance parameters of each model remain consistent with one another. Figure 8 presents a comparison of various CNN models with a batch size of 16, while Fig. 9 displays the confusion matrix for the models VGG-16 and DenseNet101, as well as VGG-19 and DenseNet201, with a batch size of 16 shown in Table 8.

**Table 6:** Various convolutional neural network model’s parameters

Model Name	Input layer size	Output layer size	layer number	Trainability (parameters)
DenseNet101	(224 × 224 × 3)	(4, 1)	101	8
Visual Geometry Group-16	(224 × 224 × 3)	(4, 1)	16	138
DenseNet201	(224 × 224 × 3)	(4, 1)	201	10.2
Visual Geometry Group-19	(224 × 224 × 3)	(4, 1)	19	143

**Table 7:** All models’ training performance with a batch size of 16

Model	No of iterations (epoch)	Loss		Rate of error	Testing accuracy (%)
		Training	Testing		
Visual Geometry Group “16”	5	0.078	0.389	0.09	93.11
	10	0.071	0.332	0.09	93.23
	15	0.068	0.274	0.08	94.11
	20	0.069	0.201	0.07	94.39
	25	0.056	0.197	0.06	94.87
DenseNet101	10	0.038	0.511	0.07	94.12
	15	0.031	0.412	0.06	94.78
	20	0.028	0.388	0.05	95.05
	25	0.025	0.361	0.04	95.15
Visual Geometry Group “19”	5	0.101	0.119	0.071	94.98
	10	0.081	0.104	0.048	95.82
	15	0.069	0.092	0.041	96.13
	20	0.033	0.081	0.036	96.87
	25	0.027	0.081	0.032	96.77
DenseNet101	10	0.059	0.079	0.05	96.79
	15	0.051	0.071	0.05	97.08
	20	0.048	0.062	0.03	98.12
	25	0.041	0.058	0.03	98.34

**Table 8:** Model-specific parameters for a confusion matrix of batch size 16

Deep learning model	Sensitivity	Specificity	Precision	Accuracy	Recall	F2 score
VGG16	94.53	95.65	89.78	95.04	93.78	93.28
DenseNet101	99.3	95.86	86.84	97.12	94.23	94.13
VGG19	95.86	99.5	99.5	99.11	98.47	97.21
DenseNet201	94.32	98.12	94.32	97.13	96.02	96.86

### Various pretrained model confusion matrices

Figure 9 displays the confusion matrices for all deep learning models with a batch size of 16. These matrices represent both accurate and inaccurate predictions equally. Each column is labeled with the class name to which it belongs, such as "normal" and "malignant." The accuracy of image classifications by a particular model can be determined from the diagonal values. This confusion matrix serves as the basis for assessing the accuracy of each model for batch sizes of 16. Figure 11 presents a graphical analysis of the accuracy of all the models. In Fig. 9, it is evident that VGG19 and DenseNet201 are the top performers in terms of accuracy obtained, achieving 99.11% and 97.13%, respectively, for a batch size of 16. These results indicate that VGG19 is the best-performing model among those tested for batch sizes of 16. Figure 12 depicts the learning rate curves for VGG19 and DenseNet201 at a batch size of 16. The learning rate curve indicates the speed at which a model learns, which can vary from slow to rapid. There is a point where the loss ceases to decrease and starts to increase as the learning rate rises. To achieve optimal results, the learning rate should be positioned to the left of the lowest point on the graph.

**Fig. 10:**
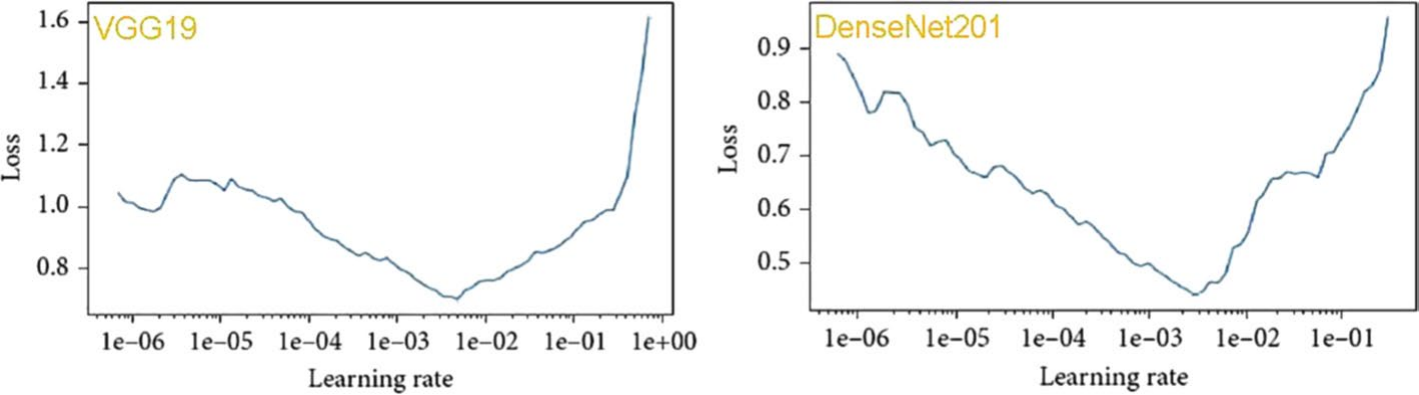
Proposed model with 16 batch sizes; learning rate versus loss curve

**Fig. 11:**
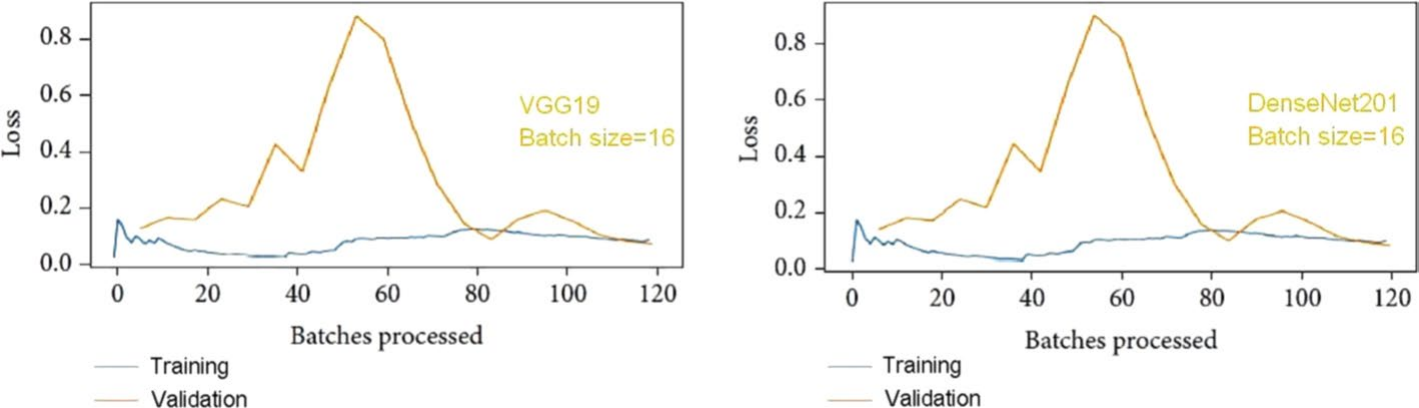
Various CNN architectures and batches processed versus a loss curve with a 16-batch size

If we examine Fig. 10 for VGG19's learning rate, we can observe that the lowest loss occurs around point 0.001. This suggests that the optimal learning rate for VGG19 should be between 0.0001 and 0.001. Similarly, the lowest loss point for DenseNet201 can be observed at 0.00001 in Fig. 10, which illustrates the learning rate. Therefore, the ideal learning rate for DenseNet201 falls between 0.000001 and 0.00001, with the loss being inversely proportional to the learning rate. Figure 11 displays the loss convergence map for the VGG19 and DenseNet201 CNN models at a batch size of 16.

The loss initially decreased as the models learned from the data, continuing until they reached a point where further improvement during training was no longer possible. Testing losses were computed for each epoch, revealing consistently small values that increased as the number of epochs progressed. Figure 11 illustrates that for a batch size of 16, both VGG19 and DenseNet201 consistently achieve their lowest loss at every epoch. Specifically, after processing 120 batches, VGG-19 exhibits lower loss than DenseNet-201. In comparison to DenseNet201, the testing and training loss for VGG19 range from 0 to 0.2, while for DenseNet201, it ranges from 0.2 to 0.4. It is evident that VGG19 outperforms DenseNet201 at a batch size of 16 in terms of training and testing loss.

### Performance metric evaluation

**Fig. 12:**
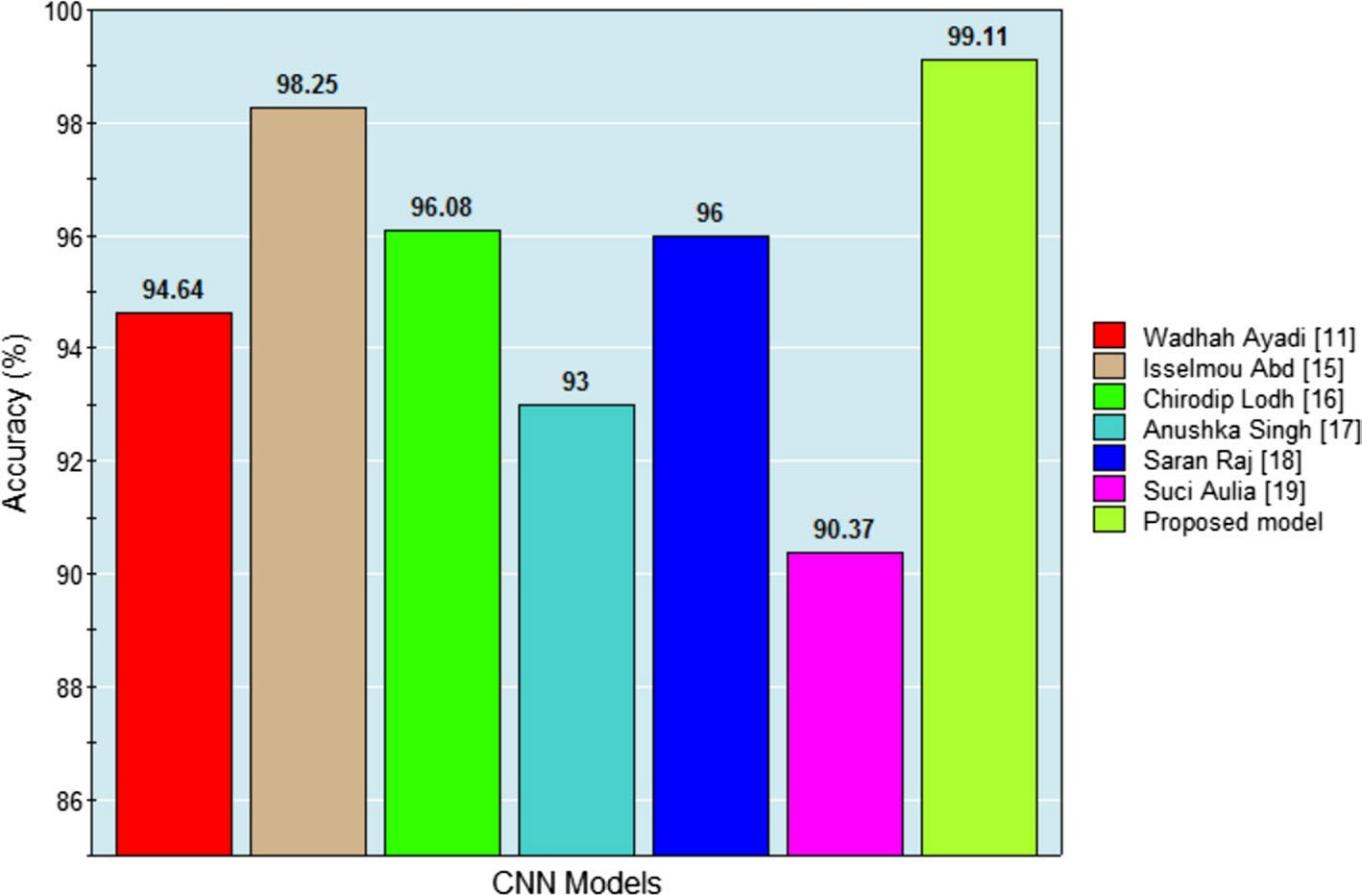
Classification accuracy comparison of proposed and existing models

As demonstrated in Table 9, the results of the proposed model are compared to those of state-of-the-art models using CT scans. Thanks to the pre-processing techniques applied to the dataset, the proposed model was able to produce a good set of results. In order to further enhance the accuracy of the proposed model, data augmentation and normalization strategies have been implemented for VGG19 and DenseNet201. The designed model achieves better results when using the ADAM optimizer with a batch size of 16. Table 9 provides a comparison of classification accuracy between the proposed model and other state-of-the-art models. The analysis in Table 9 shows that the proposed model outperforms the state-of-the-art methods in terms of all parameters, achieving a classification accuracy of 99.11%, surpassing other existing methods, despite the size of the image dataset. Figure 12 visually represents the comparison of classification accuracy between the proposed brain tumor classification model and other state-of-the-art models.

**Table 9:** Classification accuracy comparison of proposed and state-of-the-art-methods

Author	Year	Method	Dataset	Images	Accuracy outcome (%)
Isselmou Abd El Kader [15]	2021	Dilatdifferential deep-CNN architecture CNN	Tianjin Universal Centre of Medical Imaging and Diagnostic	3200	99.25
Chirodip Lodh Choudhury [16]	2020	Convolutional neural network	Kaggle	1900	96.08
Anushka Singh [17]	2020	Brilliant deep convolutional neural networks	Figshare Dataset	2100	93
Saran Raj [18]	2023	Neural Autoregressive Distribution Estimation	CE-MR brain dataset	3064	96
Suci Aulia [19]	2022	Clip Limit Adaptive Histogram Equalization	TCIA	7858	90.37
Proposed model	2023	Hybrid Deep Learning	Kaggle	2100	99.11

### Potential applications of proposed model

Deep learning-based brain tumor classification has numerous potential applications across various domains, including healthcare, medical research, and image analysis. Here are some key potential applications,

- Deep learning models can aid radiologists and clinicians in accurately diagnosing brain tumors from medical imaging data such as MRI scans. This can lead to earlier detection and better treatment planning for patients.
- Deep learning algorithms can be used to segment brain tumors from surrounding healthy tissue in medical images. This is valuable for precise surgery planning and radiation therapy. Deep learning can provide decision support for clinicians by suggesting treatment options based on a patient's tumor characteristics and medical history.
- Deep learning can assist in patient selection for clinical trials, ensuring that participants meet specific criteria related to tumor types and characteristics.

## Conclusion and future work

In this study, our aim was to thoroughly evaluate the capabilities of four powerful deep learning models: VGG16, DenseNet101, VGG19, and DenseNet201. We sought to assess their effectiveness in distinguishing malignant tumors. VGG19 and DenseNet201 emerged as top performers, particularly when used with a batch size of 16. We subjected these models to rigorous training, systematic analysis, and presented the synthesis of our findings. Furthermore, we delved into the realm of optimization to maximize the potential of the VGG19 model. By fine-tuning batch sizes, optimizing with the Adam optimizer, and adjusting the number of epochs, we achieved exceptional results. Specifically, the VGG19 model, when combined with the Adam optimizer and a batch size of 16, achieved an impressive accuracy of 99.11% and a sensitivity of 95.86%. Similarly, the DenseNet201 model, under the same conditions, delivered competitive results with an accuracy of 97.13% and a sensitivity of 94.32%. These comparative findings hold promise for providing valuable support to radiologists seeking a reliable second opinion tool or simulator, potentially offering a cost-effective alternative in the field of tumor diagnosis. Our central mission throughout this study has been to pioneer methods for early malignancy detection, envisioning a tool that could empower radiologists in their diagnostic endeavors. The insights gained from this research contribute significantly to the advancement of precision-driven diagnostic models within the realm of deep learning. However, it is important to acknowledge a notable limitation in our research. We limited our training and testing efforts exclusively to a single axial dataset comprising brain malignant samples. Recognizing the potential for greater generalization and robustness, we anticipate future expansions of our model to include coronal and sagittal datasets in both the training and testing phases. Additionally, our ongoing pursuit of excellence motivates us to explore a wide array of pretrained models and innovative optimization strategies, promising to further enhance the efficiency and reliability of our model.

## Data Availability

The datasets used during the current study are available from the corresponding author on reasonable request.
